# Chagas Disease in northern Minas Gerais: Clinical and epidemiological features of patients at a pioneering specialized outpatient service

**DOI:** 10.1371/journal.pntd.0014488

**Published:** 2026-07-06

**Authors:** Luciano Freitas Fernandes, Gláucia Rejanny Teixeira Fernandes, Ester Cerdeira Sabino, Sâmara Fernandes Leite, Mayra Domingues Cardoso, Ana Julia Torres Bonfim Rocha, Ana Clara de Jesus Santos, Ana Beatriz Cardoso Sena, Dardiane Santos Cruz, Ariela Mota Ferreira, Israel Molina

**Affiliations:** 1 University of Santa Casa de Montes Claros, Montes Claros, Minas Gerais, Brazil; 2 Graduate Program in Health Sciences, State University of Montes Claros (UNIMONTES), Montes Claros, Minas Gerais, Brazil; 3 Institute of Tropical Medicine, University of São Paulo (USP), University of São Paulo Institute, São Paulo, São Paulo, Brazil; 4 State University of Montes Claros, Montes Claros, Minas Gerais, Brazil; 5 Department of Infectious Diseases, Vall d’Hebron University Hospital, PROSICS Barcelona; Centro de Investigación Biomédica en Red de Enfermedades Infecciosas (CIBERINFEC), Instituto de Salud Carlos III, Madrid, Spain; Fundacao Oswaldo Cruz, BRAZIL

## Abstract

**Introduction:**

Chagas Disease (CD) remains a persistent public health problem in Brazil, particularly in historically hyperendemic regions such as northern Minas Gerais. Despite the sustained burden of transmission and chronic disease, access to specialized care for CD continues to be limited. This study aimed to describe the clinical and epidemiological profile of patients attending a newly established specialized CD outpatient clinic and to contextualize its role within the regional hyperendemic scenario.

**Methods:**

A descriptive cross-sectional assessment was conducted using routinely collected clinical information from medical records of patients who attended the service between October 2022 and September 2024. Data extraction combined review of paper and electronic records with verification through on-site clinical documentation.

**Results:**

Over the two-year period, the clinic experienced a substantial increase in demand. In the first year, 274 patients received care, whereas in the second year this number rose to 657—an increase of 239.7%. Overall, 931 patients attended the service. The majority of patients were female, with a mean age of 58 (± 11.1) years, and had the indeterminate form of CD (47.2%). A total of 630 patients (68.5%) underwent etiological treatment for the disease. Among these, 309 received prescriptions directly from the outpatient clinic, and 208 out of them (67.3%) experienced adverse drug reactions. The clinic provides care for the northern macro-region of Minas Gerais, encompassing 86 municipalities: 45 (52%) of these referred patients during the study period. Referral patterns highlighted contrasting municipal profiles: Montes Claros was the largest source of patients.

**Conclusion:**

The findings demonstrate the growing demand for specialized CD care and underscore the importance of dedicated services in regions where primary care lacks structured clinical pathways for CD management. The clinic offers not only access to etiological treatment and longitudinal follow-up but also a reproducible model for expanding equitable care for neglected diseases in other endemic settings.

## Author summary

Chagas Disease remains a significant public health problem in northern Minas Gerais, Brazil, a region where access to specialized healthcare services is still limited. To address this gap, a specialized Chagas Disease outpatient clinic was recently implemented. In this study, we describe the clinical and epidemiological profile of patients treated at this clinic. We analyzed medical records of patients treated between October 2022 and September 2024. During this period, 931 patients were treated, with a substantial increase in demand between the first and second years of operation. Patients came from more than half of the municipalities in the northern macro-region of Minas Gerais, highlighting the broad geographic reach of the service. Our findings demonstrate a high and growing demand for specialized Chagas Disease care and emphasize the importance of dedicated outpatient services to improve access to diagnosis, treatment, and clinical follow-up. This outpatient clinic represents a potentially scalable and replicable model for strengthening care for Chagas Disease in other endemic regions.

## Introduction

Chagas Disease (CD), caused by *Trypanosoma cruzi*, is recognized by the World Health Organization (WHO) as one of the thirteen most neglected tropical diseases and remains a major public health concern across Latin America [1]. In Brazil, CD is considered not only a biomedical issue but also one of the country’s most pressing socio-medical challenges [2].

The disease is potentially lethal: up to 42% of patients may develop Chronic Chagas Cardiomyopathy (CCC), often decades after the initial infection [3]. This highlights the urgent need for systematic medical attention. Yet, limited awareness and insufficient training on CD and CCC among healthcare professionals frequently result in inadequate follow-up, management, and treatment [4–6]. A national review of ten years of mortality data reported around 5,000 deaths attributable to CD, underscoring its persistent burden [7].

The state of Minas Gerais represents one of the country’s most relevant endemic regions [8]. It accounts for the highest number of CD-related deaths nationwide and ranks among the three Brazilian states with the greatest vulnerability to chronic CD [9]. Within the state, the North of Minas region is particularly critical: it reports nearly half of all chronic cases in Minas Gerais and presents the second-highest CD Vulnerability Index (CDVI) at both state and national levels [10,11]. Current prevalence estimates remain strikingly high, reaching up to 9.2% in some areas, nearly five times the state average of 2.1% [12,13].

In the absence of a national policy to structure care for CD patients, primary healthcare (PHC) has carried the responsibility of managing these individuals without the support of an organized referral network [12]. Physicians working in endemic regions have highlighted critical challenges: insufficient training for CD management, uncertainties regarding antiparasitic treatment in the chronic phase, limited access to specialized outpatient care, and the tendency of patients to underestimate the disease, delaying care-seeking [5,14,15].

Until 2022, the northern macro-region of Minas Gerais, encompassing 86 municipalities, lacked any specialized outpatient service for CD within the Brazilian Unified Health System (SUS). Given the strong link between CD and social vulnerability, implementing a structured model of care has the potential to reduce long-standing health inequities for patients, their families, and communities, while addressing the broader neglect of this disease.

In response, a specialized outpatient clinic for CD was established to serve as a regional referral center, providing diagnosis, follow-up, and treatment for patients across the macro-region. The present study aims to describe the clinical and epidemiological profile of patients attending this pioneering service, while situating it within the hyperendemic context of northern Minas Gerais.

## Methods

### Ethic statement

The study was approved by the Ethics Committee of the State University of Montes Claros (approval no. 7.076.797) and conducted in accordance with Resolution 466/2012 of the Brazilian National Health Council. As this study used secondary data, a waiver of informed consent was requested.

We conducted a descriptive cross-sectional study based on medical records of patients attending the CD outpatient clinic over a two-year period (October 2022–September 2024). Information was extracted from both paper-based and electronic records provided by the service administration and complemented by on-site professional documentation. The CD outpatient clinic serves the northern macro-region of Minas Gerais, an endemic area encompassing 86 municipalities. The dataset generated is available at the SciELO Data repository.

Clinical care at the clinic follows standardized operating procedures covering patient referral, diagnosis, treatment, and follow-up. Referral from primary healthcare (PHC) is mandatory to ensure continuity of care. Patients are required to present two positive serological tests based on different diagnostic methods, in accordance with the national Clinical Protocol and Therapeutic Guidelines (PCDT) for CD [14], and notification in the national E-SUS system, as CD has been a compulsory notifiable disease in Brazil since 2018. At the clinic, patients undergo specialist consultations (infectious disease and cardiology), vital sign assessment, epidemiological interview, and nursing consultation.

Variables analyzed were grouped into sociodemographic and clinical characteristics. Sociodemographic variables included sex (female vs. male), age, self-reported skin color (white, yellow, mixed-race, or Black), and membership in traditional communities. Clinical variables included: family history of prior CD screening, clinical form of CD (cardiac, digestive, cardiodigestive, or indeterminate), mean age by clinical form, hypertension, diabetes, history of stroke, presence of pacemaker, obtained from reports provided by the patients, (any minor or major change is considered to be altered), left ventricular ejection fraction, and body mass index (BMI) [16]. Among the group of patients treated at the outpatient clinic, the variables related to drug reactions were collected.

For the classification of cardiac involvement, a 12-lead ECG was used, applying the criteria established in the Brazilian Society of Cardiology guidelines [16]. The digestive form was defined by evidence of megaesophagus and/or megacolon detected through plain radiography or digestive endoscopy. Patients classified as indeterminate were those in the chronic phase of CD without specific clinical syndromes, with normal ECG, chest X-ray, and esophageal and colonic studies [17].

We also recorded medication classes used, including antihypertensives, angiotensin-converting enzyme inhibitor/angiotensin receptor blocker (ACE inhibitors/ARB), diuretics, antiarrhythmics, alpha/beta-blockers, statins, hypoglycemic agents, antidepressants, anticoagulants, calcium channel blockers, vasodilators, and angiotensin receptor–neprilysin inhibitor (ARNI).

In the outpatient clinic, a standardized template is used to collect patient information, except for medical progress notes, which facilitates standardization and, consequently, improves data quality. The researchers responsible for data collection were previously trained and calibrated regarding data extraction procedures. In cases where the information found in the records did not follow the expected standard or raised interpretative uncertainties, the records were reviewed and discussed jointly by all authors to ensure greater consistency and reliability of the collected data.

To assess contextual factors, we used the Chagas Disease Vulnerability Index (CDVI) [18] and the 2010 Municipal Human Development Index (MHDI) [19]. The CDVI, developed by the Brazilian Ministry of Health in 2022, identifies areas with greater potential morbidity and mortality from chronic CD in association with healthcare access barriers. It comprises three sub-indices: (i) epidemiological indicators directly related to chronic CD; (ii) indicators of conditions arising from disease progression; and (iii) indicators of healthcare access. Scores range from 0 to 1, with higher values indicating greater vulnerability. The MHDI is the geometric mean of income, education, and life expectancy indices, categorized as low, medium, high, or very high.

Descriptive analyses were performed for all individual variables, with absolute and relative frequencies reported. For age, we also calculated mean and standard deviation. Missing data were treated as losses in the analysis. Spearman’s correlation was applied to assess the relationship between the number of clinic visits per municipality and both CDVI and MHDI scores. Analyses were conducted using Predictive Analytics Software (PASW/SPSS) version 18.0.

## Results

The CD outpatient clinic was established in October 2022 at the Tancredo Neves Specialty Center (CAETAN), affiliated with the Clemente de Faria University Hospital of the State University of Montes Claros (Unimontes). Its implementation was driven by researchers from the SaMi-Trop project, representing a social return from research on CD conducted in the region since 2014 [20]. The initiative involved a partnership between Unimontes, its university hospital, the Minas Gerais State Health Department, and the Municipal Health Department of Montes Claros. The service was formally approved during a bipartite inter-management committee (CIB) meeting with participation from all municipalities in the region, where patient referral and care flows were defined.

For the care of patients with CD, a standard operating procedure was developed for scheduling, diagnosis, treatment and management. At the scheduling, three documents are required. 1) a referral from primary health care, aimed at maintaining the patient’s linkage with this level of care; 2) two positive serological tests using different diagnostic methods, as recommend by the PCDT [14]; 3) a recorded compulsory notification form. After the initial visit, the necessary complementary tests are requested to assess the clinical form and progression of CD. The scheduling of follow-up visits is determined according to the patient’s access to these tests within the health system, as appointment booking is managed by primary health care, which may influence the interval between visits.

**Fig 1:**
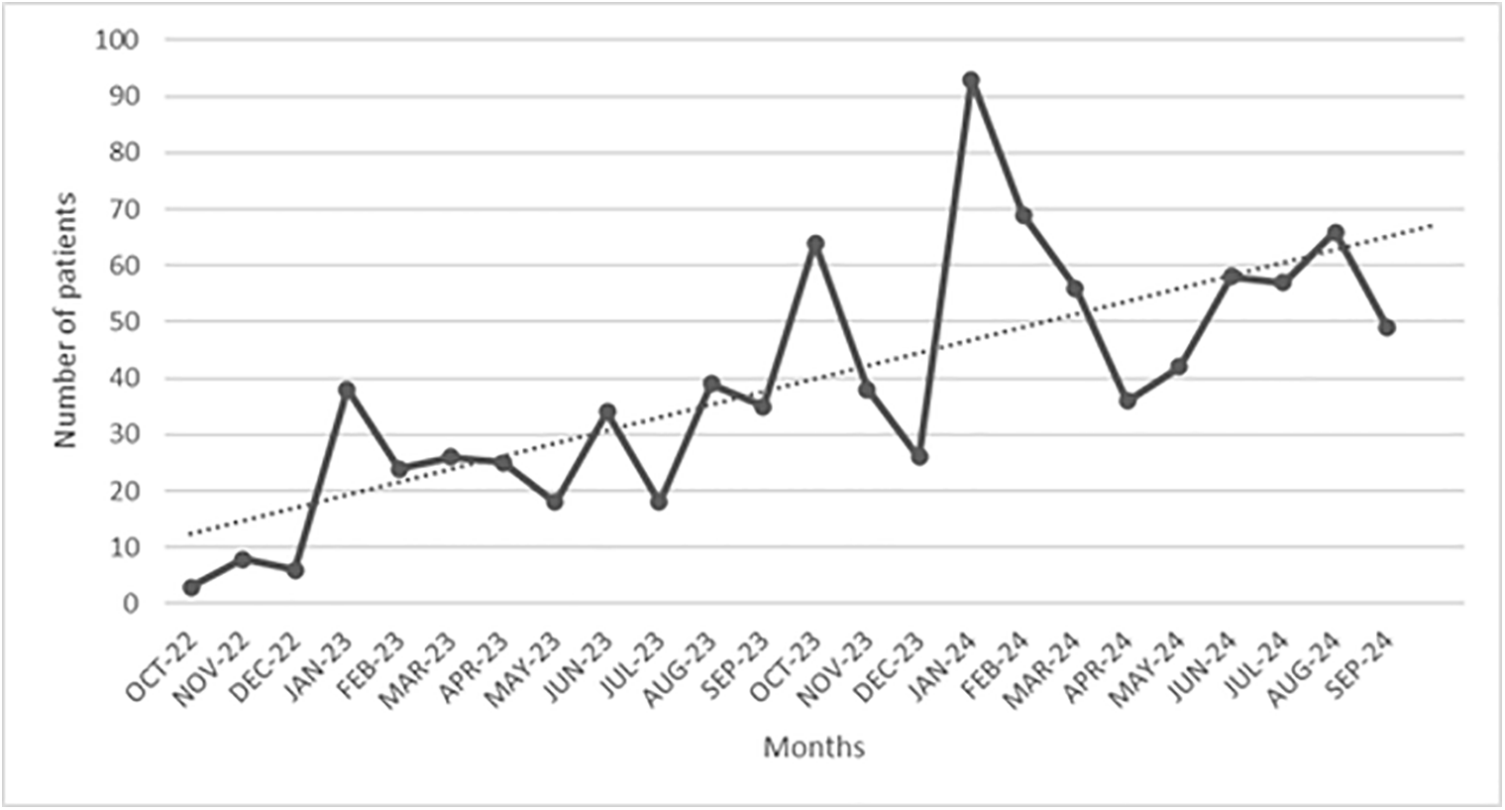
Number of patients per month at Chagas Disease outpatient clinic. Minas Gerais. Brazil, 2023-2024.

**Fig 2:**
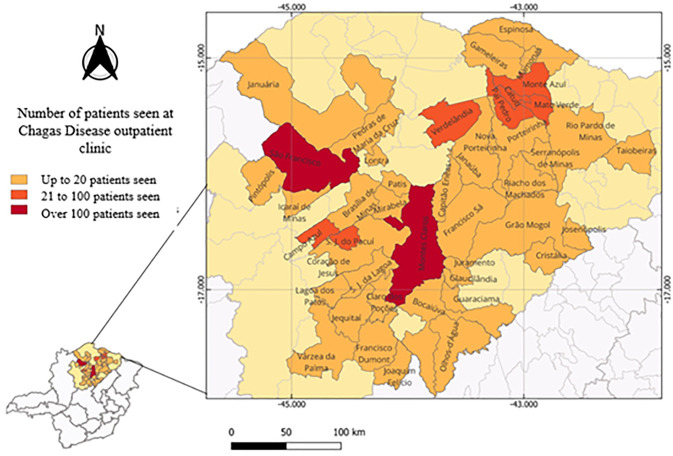
Number of patients referred to Chagas Disease outpatient clinic. Minas Gerais, Brazil, 2023-2024. Cartographic elaboration: Ana Clara de Jesus Santos (2025). Sources: municipal and state boundaries (IBGE, 2023). Geographic coordinate system, datum SIRGAS 2000. Source: Malha municipal e unidades federativas do Brasil (shapefile), disponibilizadas pelo IBGE (2023). Available:https://www.ibge.gov.br/geociencias/organizacao-do-territorio/malhas-territoriais/15774-malhas.html.

Over the two-year evaluation period, a steady increase in service utilization was observed. In the first year, 274 patients with CD were seen, while in the second year this number more than doubled (+239.7%), reaching 657 patients (Fig 1). The clinic provides care for the northern macro-region of Minas Gerais, comprising 86 municipalities. During the study period, 45 (52%) municipalities referred patients for care (Fig 2).

Among them 931 patients attended over the two-year period, the majority (64%) were female, with a mean age of 58 (± 11.1) years and self-reported Brown skin color. Among the medical records evaluated in this study, information was obtained regarding treatment with BZN, whether prior at some point in life or conducted in an outpatient setting. Among the patients evaluated, 630 (68.5%) had received benznidazole treatment at some point in their lives, with a mean treatment duration of 59 days (±24.1; range: 1–180 days). Most were female, with a mean age of 60.8 (± 12.8) years and self-reported Brown skin color. Among treated patients with clinical form data available, the indeterminate form was most frequent (n = 260; 51.7%) (Table 1). It was observed that 94 (14.9%) patients completed at least 30 days of treatment, and 402 (63,8%) completed 60 days of treatment, as recommended by the PCDT.

**Table 1:** Descriptive and bivariate analysis of the individual and clinical characteristics of patients with Chagas disease, according to antiparasitic treatment at some point in their lives (n = 931).

*Characteristics*	*Mean*	*Descriptive* n (%)	*Bivariate* *n (%)*		
			*Untreated*	*Treated*	
***Sociodemográficas***					
Sex					
Female		597 (64.1)	184 (31.2)	406 (68.8)	0.770
Male		334 (35.9)	106 (32.1)	224 (67.9)	
Age					
Mean	58.7 (± 11.1)		57.60 (±10)	60.85 (±12.8)	≤0.001
Self-reported skin color*					
White		135 (15.6)	49 (36.8)	84 (63.2)	0.069
Yellow		21 (2.4)	2 (9.5)	19 (90.5)	
Brown		565 (65.2)	171 (30.4)	392 (69.6)	
Black		146 (16.8)	42 (28.8)	104 (71.2)	
Belongs to tradicional communities					
Indigenous		2 (0.2)	0 (0.0)	2 (0.3)	0.108
Quilombola		39 (4.2)	7 (2.4)	32 (5.1)	
None		890 (95.6)	283 (97.6)	596 (94.6)	
***Clinical characteristics***					
Family members previously screened for CD*					
No		370 (43.7)	116 (31.4)	253 (68.6)	0.626
Yes		475 (56.3)	114 (30.7)	325 (69.3)	
Clinical form of CD*					
Indeterminate		337 (47.2)	75 (22.4)	260 (77.6)	
Cardiac		329 (46.1)	113 (34.5)	215 (65.5)	
Digestive		29 (4)	12 (44.4)	15 (55.6)	
Cardiodigestive		18 (2.5)	6 (33.3)	12 (66.7)	
Mean age according to the clinical form of CD*					
Indeterminate	59.1 (±10.6)		55.9 (±10.7)	59.6 (±14.2)	0.002
Cardiac	61.7 (±11.2)		58.4 (±9.5)	61.6 (±12.3)	
Digestive	56.1 (±11.7)		59.5 (±12.2)	63.2 (±8.5)	
Cardiodigestive	63.3 (±11.9)		61.7 (±10.7)	66.5 (±14.7)	
Arterial hypertension*					
No		369 (43)	93 (25.2)	276 (74.8)	0.005
Yes		490 (57)	165 (34.1)	319 (65.9)	
Diabetes*					
No		753 (87.5)	225 (30.1)	522 (69.9)	0.774
Yes		108 (12.5)	34 (31.5)	74 (68.5)	
Stroke history*					
No		511 (95.2)	146 (28.7)	362 (71.3)	0.006
Yes		26 (4.8)	14 (53.8)	12 (46.2)	
Pacemaker use					
No		673 (95.2)	174 (26.0)	494 (74.0)	0.002
Yes		34 (4.8)	17 (50.0)	17 (50.0)	
Mean ejection fraction*					
	62.5 (± 11.5)		64.1 (±10.3)	59.0 (±13.0)	≤0.001
Average body mass index *					
	26.7 (± 5.2)		26.9 (±5.1)	26.3 (±5.4)	0.081

*Variation because of missing information.

For treatment prescription, the outpatient clinic follows the criteria established by the PCDT, which include patients under 50 years of age with the indeterminate form of the disease or mild cardiac involvement. However, for individuals over 50 years, the benefit is considered more uncertain, therefore, the decision regarding treatment is shared between doctor and patient [14].

**Fig 3:**
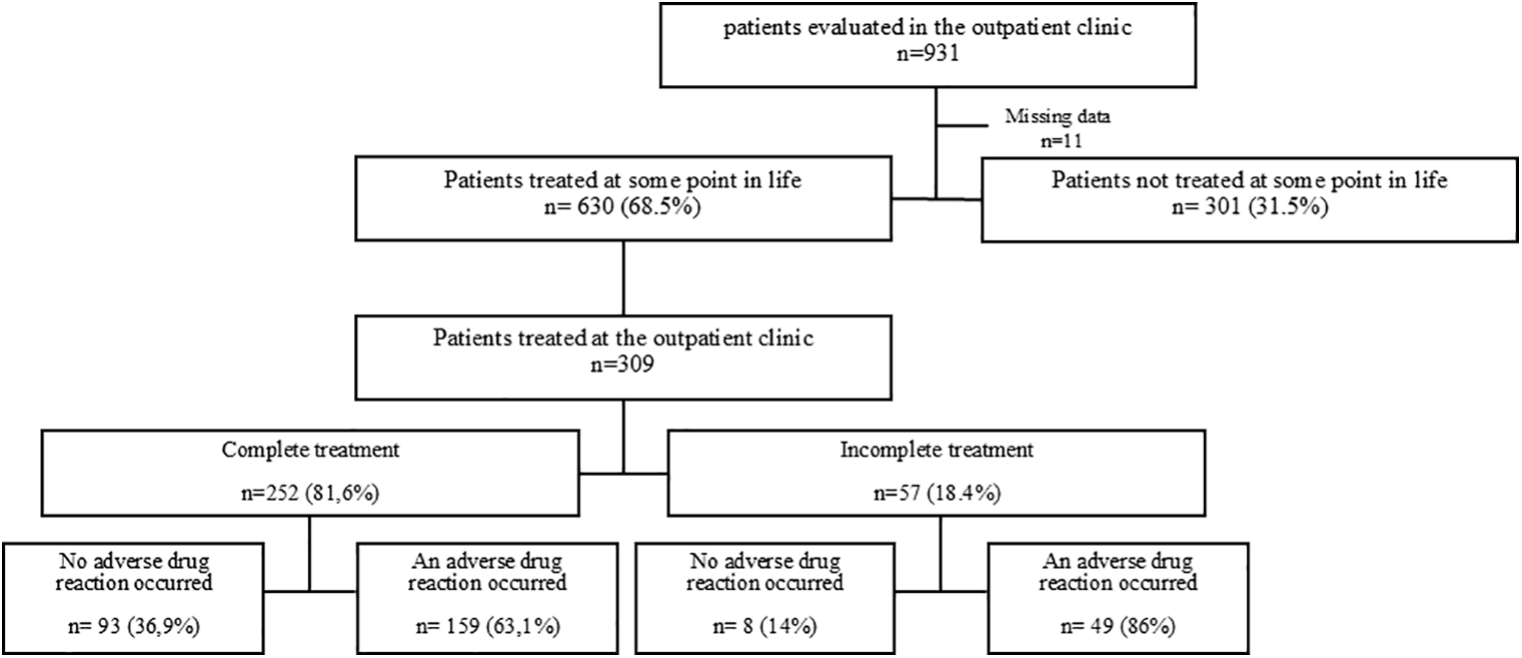
Flowchart of patients with Chagas disease evaluated in the outpatient clinic, according to lifetime etiological treatment status, treatment performed at the study outpatient clinic, treatment completion, and occurrence of adverse drug reactions. Minas Gerais, Brazil, 2023–2024.

Of the 630 patients who had received benznidazole treatment at some point in their lives, 309 were treated at the outpatient clinic evaluated in this study (Fig 3). Of these, 252 (81.6%) completed the prescribed treatment and 208 (67.3%) patients experienced adverse drug reactions. Treatment discontinuation was more frequent among older patients and those experiencing adverse drug reactions, particularly neurological events (Table 2).

**Table 2:** Descriptive and bivariate analysis of individual characteristics and adverse reactions of patients with Chagas disease treated in a specialized outpatient clinic, according to the completion of antiparasitic treatment (n = 309).

*Characteristics*	*Mean*	*Descriptive* n (%)	*Treatment* *n (%)*		*p-value*	*OR (IC95%)*
			*Completed*	*Incomplete*		
***Sociodemográficas***						
Sex						
Female		189(61.2)	148 (78.3)	41 (21.7)	0.065	1
Male		120 (38.8)	104 (86.7)	16 (13.3)		0.555 (0.296-1.043)
Age						
Mean	56.8 (±10.1)		56.3 (±10.3)	59.2 (±9.1)	**0.052**	1.030 (1.000-1.062)
Self-reported skin color*						
White		48 (15.7)	39 (81.3)	9 (18.8)	0.580	1
Yellow		10 (3.3)	7 (70)	3 (30)		1.857 (0.297–11.635)
Brown		206 (67.3)	171 (83)	35 (17)		0.887 (0.374–2.104)
Black		42 (13.6)	32 (76.2)	10 (23.8)		1.354 (0.455–4.029)
Had adverse drug reactions						
No		101 (32.7)	93 (92.1)	8 (7.9)	**0.001**	1
Yes		208 (67.3)	159 (76.4)	49 (23.6)		3.583 (1.626-7.893)
Dermatological adverse reaction n = 208						
No		82 (39.4)	65 (79.3)	17 (20.7)	0.517	1
Yes		126 (60.6)	95 (75.4)	31 (24.6)		1.248 (0.638-2.439)
Gastrointestinal adverse reaction n = 208						
No		112 (53.8)	84 (75)	28 (25)	0.477	1
Yes		96 (46.2)	76 (79.2)	20 (20.8)		0.789 (0.411-1.516)
Neurological adverse reaction n = 208						
No		162 (7.9)	132 (81.5)	30 (18.5)	**0.003**	1
Yes		46 (22.1)	28 (60.9)	18 (39.1)		2.829 (1.387-5.768)

To minimize treatment discontinuation due to adverse drug reactions, the clinic implemented a follow-up protocol. Patients were carefully counseled on potential side effects, received weekly telephone follow-up, and were managed promptly with symptomatic treatment or temporary treatment interruption when necessary. Scheduled follow-up visits were conducted at 30 days and at the end of treatment, including clinical evaluation and laboratory monitoring with complete blood count, renal function tests, and assessment of liver enzymes [14].

**Fig 4:**
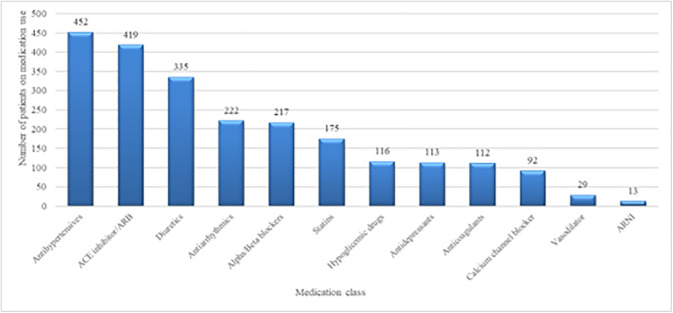
Number of patients with Chagas Disease using medications, according to drug class. Minas Gerais, Brazil, 2023-2024. *inhibitor/angiotensin receptor blocker (ACE inhibitors/ARB). *angiotensin receptor–neprilysin inhibitor (ARNI).

Medication use was evaluated, by therapeutic class, among patients seen in the outpatient clinic, showing antihypertensives as the most frequently prescribed (Fig 4). Notably, patients often used more than one class concurrently.

Among referring municipalities, Montes Claros had the highest number of patients, despite being among those with the lowest CDVI and the highest Municipal Human Development Index (MHDI). Conversely, São Francisco stood out as the second largest source of referrals, despite ranking among the 11 municipalities with the highest CDVI and among the 14 with the lowest MHDI (Table 3). Correlation between the number of referrals and CDVI (r = 0.022; p = 0.885) or MHDI (r = 0.007; p = 0.966) was weak.

**Table 3:** Descriptive values and classification of the MHDI and the Chagas Disease vulnerability index of the municipalities that referred patients to the outpatient clinic. (n = 45).

Municipalities	MHDI*	MHDI Classification	Chagas Disease Vulnerability Index
Bocaiuva	0.700	High MHDI	0.152
Brasília de Minas	0.692	Mean MHDI	0.176
Campo Azul	0.621	Mean MHDI	0.121
Capitão Enéas	0.639	Mean MHDI	0.133
Catuti	0.621	Mean MHDI	0.314
Claro dos Poções	0.670	Mean MHDI	0.204
Coração de Jesus	0.642	Mean MHDI	0.109
Cristália	0.583	Low MHDI	0.143
Espinosa	0.627	Mean MHDI	0.043
Francisco Dumont	0.625	Mean MHDI	0.172
Francisco Sá	0.603	Mean MHDI	0.213
Gameleiras	0.650	Mean MHDI	0.266
Glaucilândia	0.679	Mean MHDI	0.193
Grão Mogol	0.604	Mean MHDI	0.248
Guaraciama	0.677	Mean MHDI	0.199
Icaraí de Minas	0.624	Mean MHDI	0.288
Janaúba	0.696	Mean MHDI	0.146
Januária	0.658	Mean MHDI	0.091
Jequitaí	0.643	Mean MHDI	0.188
Joaquim Felício	0.637	Mean MHDI	0.112
Josenópolis	0.617	Mean MHDI	0.497
Juramento	0.669	Mean MHDI	0.095
Lagoa dos Patos	0.634	Mean MHDI	0.106
Lontra	0.646	Mean MHDI	0.216
Mamonas	0.618	Mean MHDI	0.118
Mato Verde	0.662	Mean MHDI	0.144
Mirabela	0.665	Mean MHDI	0.177
Monte Azul	0.659	Mean MHDI	0.157
Montes Claros	0.724	High MHDI	0.089
Nova Porteirinha	0.641	Mean MHDI	0.136
Olhos D’Água	0.626	Mean MHDI	0.077
Pai Pedro	0.669	Mean MHDI	0.108
Patis	0.614	Mean MHDI	0.175
Pedras de Maria da Cruz	0.614	Mean MHDI	0.250
Pintópolis	0.594	Low MHDI	0.076
Porteirinha	0.651	Mean MHDI	0.053
Riacho dos Machados	0.627	Mean MHDI	0.190
Rio Pardo	0.624	Mean MHDI	0.150
São Francisco	0.660	Mean MHDI	0.201
São João da Lagoa	0.634	Mean MHDI	0.128
São João do Pacuí	0.625	Mean MHDI	0.095
Serranópolis de Minas	0.633	Mean MHDI	0.128
Taiobeiras	0.670	Mean MHDI	0.056
Várzea da Palma	0.666	Mean MHDI	0.150

* Source: Human Development Atlas. http://www.atlasbrasil.org.br/2013/pt/o_atlas/idhm/.

## Discussion

This study describes the clinical and epidemiological profile of patients with Chagas Disease (CD) attended at the first specialized outpatient clinic within the SUS in a hyperendemic region of Brazil. The growing number of referrals over the two-year period highlights the urgent need for this service. In total, 931 patients were evaluated, coming from 45 municipalities.

The implementation of a specialized outpatient clinic for CD is aligned with SUS policy, which emphasizes equity in health not only in terms of generalized access but also in addressing the real endemic needs of specific populations. In this case, the service responds to the demands of a population that has long remained invisible within the health system, lacking systematic registration in primary healthcare and without structured follow-up. These patients generate a considerable burden for the public health system and remain largely underserved by the private sector [21].

Descriptions of specialized care models for Chagas Disease are scarce in the literature. In Guatemala, the implementation of a care model supported by the Drugs for Neglected Diseases (DNDi) enabled the integration of a specialized clinic into the local health network, offering comprehensive care, including etiological treatment and longitudinal clinical follow-up. This model prioritized women of reproductive age and pregnant women, who accounted for more than 76% of the treatments provided [22]. In Brazil, particularly in Minas Gerais, region characterized by high vulnerability to the disease [18], no records were identified in the literature, nor in national health facility databases, describing the existence of structured specialized services for the management of CD [23]. In this context, the present outpatient clinic represents a pioneering initiative, as it implements and describes a care model in an endemic region. Nonetheless, the consolidation and ampliation of such models remain subject to significant challenges. The principal challenges include the lack of qualified human resources, gaps in integration across different levels of care, including coordination between healthcare service delivery and epidemiological surveillance, and limited structural funding [24].

The estimated population of the northern region of Minas Gerais is nearly 1.7 million [25], with recent studies suggesting a CD prevalence of up to 9.2% [12], equivalent to approximately 150,000 carriers. Given the clinic’s current monthly capacity of around 50 patients, it is clear that the service is still far from meeting the total demand, particularly considering the need for periodic follow-up visits. Moreover, only about half of the municipalities in the macro-region referred patients during the study period, suggesting persistent inequalities in local health service management, logistical barriers, or limited awareness of the specialized service. Therefore, the growing demand confirms a substantial hidden burden of CD, with the clinic addressing only a fraction of the need.

The sociodemographic characteristics of the patients are consistent with prior studies. The majority were women, which may reflect the tendency of women to seek healthcare more frequently than men, particularly for preventive services [26]. The mean age of 58 years reflects a cohort that was exposed to transmission before the establishment of effective vector control measures in the 1970s [27]. However, the fact that the youngest patient is only 4 years old may suggest congenital or even vector-borne transmission [13]. The predominance of black and mixed-race patients, along with the presence of quilombola communities, reinforces the disproportionate burden of CD on socially vulnerable populations [28,29].

With respect to clinical forms, the indeterminate and cardiac forms predominated, consistent with prior epidemiological descriptions [16], highlighting the importance of systematic monitoring. Hypertension was the most frequent comorbidity, compounding the clinical complexity of this population [3].

Etiological treatment with benznidazole was prescribed to 68.5% of patients, yet nearly half of them received treatment only after referral to the outpatient clinic, underscoring the limitations of primary care in initiating therapy. In Brazil, benznidazole (BZN) is currently the only available drug for the treatment of CD, and guidelines recommend that therapy should be initiated at the primary care level. In practice, however, this is not consistently implemented. Almost half of the patients who received treatment did so only after being referred to a specialized outpatient clinic. Importantly, most patients had already been diagnosed with CD, and according to national guidelines [14], could have been identified and treated earlier. These findings suggest a relevant gap between recommendations and routine practice, which limits timely access to treatment for many patients.

The treatment with benznidazole is associated with a high rate of adverse drug reactions (44.1%- 95% CI 37.2–51.2) and of treatment discontinuations (11.4%-95% CI 8.5–14.5) [30]. The clinic’s structured protocol for monitoring treatment proved crucial in minimizing treatment abandonment.

A key innovative feature of this service is the structured follow-up protocol for benznidazole treatment, combining patient education, weekly calls, prompt clinical management, and scheduled visits. This low-cost, patient-centered adherence strategy not only improved treatment completion but also helped reduce structural inequities by securing effective care for socially vulnerable groups.

This study also has limitations. The reliance on compulsory notification through primary healthcare as a prerequisite for referral may have contributed to underreporting and left many patients uncounted and untreated. Furthermore, only 52% of municipalities referred patients, reflecting uneven health service organization and awareness across the region.

The specialized CD outpatient clinic described here highlights the need for dedicated services for neglected diseases. In the absence of systematic clinical pathways in primary care, such services are essential for providing comprehensive patient care. Their importance lies not only in facilitating etiological treatment but also in expanding follow-up and promoting equity in healthcare delivery for a historically neglected disease. As the first large-scale documented experience of its kind in that hyperendemic region, it offers a replicable framework for other endemic regions, providing both a pioneering local solution and an internationally relevant model for neglected diseases.
