# Supplementary material for: Chagas Disease in northern Minas Gerais: Clinical and epidemiological features of patients at a pioneering specialized outpatient service
Source: PLoS Negl Trop Dis. 2026 Jul 6;20(7):e0014488. doi: 10.1371/journal.pntd.0014488 (PMC13354082; doi:10.1371/journal.pntd.0014488)
Supplement: Attachment — Submitted filename: Response to Reviewers.docx [file pntd.0014488.s003.docx]

Dear Editors and Reviewers of PLOS Neglected Tropical Diseases,

We would like to thank you for the careful evaluation of our manuscript. We have fully addressed the suggested revisions and hope that the revised version now meets the publication criteria of the journal.

Below, we describe the comments and requests presented by each reviewer, followed by the authors’ responses and considerations. All modifications made to the manuscript have been highlighted in the text in order to facilitate their identification.

| **Comments** | **Author´s answers** |
| --- | --- |
| Journal Requirements:    1) Please ensure that the Title in your manuscript file and the Title provided in your online submission form are the same. | We checked it. Thank you. |
| **Results**  Reviewer #2: The direction of the odds ratios presented in Table 2 is unclear. For some variables the reported ORs appear to model treatment completion, whereas for adverse drug reactions the ORs appear to model treatment discontinuation. The authors should clarify the outcome definition used in the logistic regression analyses and verify the consistency of the reported estimates." | We thank the reviewer for this important observation. We revised Table 2 to clarify the reference categories and the direction of the logistic regression analyses. In all logistic regression models, treatment incompletion was defined as the dependent outcome. We also standardized the presentation of the reference categories throughout the table. |
| 1. “Medium” vs. “Mean” in table headers (Tables 1 and 2). In my original review, I explicitly requested that “Medium 58.7” be replaced by “Mean 58.7” (or “Median” if applicable) throughout the tables. The author response letter acknowledges this request. However, the column header in both Table 1 (“Mean Descriptive” column reads “Medium”) and Table 2 (“Age Medium 56.8 ±10.1”) still uses the incorrect term. “Medium” is not a recognized statistical descriptor; “Mean” is required. This is a straightforward typographical correction that must be made. | We apologize. Indeed, the incorrect term had still been maintained in the variable description. We have corrected it. In the table column, however, we maintained the terminology as requested. |
| 2. Decimal formatting inconsistency in Table 2. The request to ensure consistent decimal formatting across the manuscript (period separator, not comma) was acknowledged but not fully implemented. Table 2 of the original submission, now integrated into the main manuscript, continues to use comma notation in percentage values (e.g., 64,1%; 65,2%; 16,8%), whereas the revised text and Table 1 use period notation. All numerical values must use a uniform period decimal separator throughout. | 2. We thank the reviewer for the careful review. Table 2 from the original submission should not have been included in the main manuscript. We have now submitted the corrected version in order to avoid this issue. We apologize for the oversight and will be more attentive during the submission process to ensure that only the current and corrected documents are included. In addition, we carefully reviewed the manuscript and made the necessary corrections. |
| 3. Logical inconsistency between ECG findings and classification of the indeterminate form. The revised manuscript states that 69% of patients presented ECG abnormalities, with the parenthetical clarification that “any minor or major change is considered to be altered” (Discussion, R1). The manuscript simultaneously reports that the indeterminate form was the most prevalent clinical classification (47.2%). This juxtaposition creates a direct logical contradiction with the diagnostic criteria the authors themselves cite: Reference 17 (2nd Brazilian Consensus on Chagas Disease, 2015) defines the indeterminate form as requiring a normal ECG, normal chest X-ray, and normal esophageal and colonic studies. If the ECG criterion used in this study departs from the consensus, for instance, by considering only major changes as defining cardiac involvement, this must be explicitly stated in the Methods. As currently written, readers are left with an unexplained inconsistency: nearly half the cohort is classified as indeterminate, yet most of the same cohort has an abnormal ECG by the study’s own definition. The authors must add a clarifying sentence in the Methods section specifying which ECG alterations were used to reclassify a patient from indeterminate to cardiac form, with reference to the applicable guideline | We thank the reviewer for this important observation and agree that the information, as previously presented, could generate an apparent inconsistency for the reader. Since this study was conducted using secondary data obtained from medical records and routine outpatient care, there was no standardized reassessment or calibration among investigators regarding the interpretation of electrocardiographic findings and the clinical classification of patients. Although the outpatient clinic follows recommendations from official national guidelines, the classification of the cardiac form in clinical practice involves a broader and more rigorous clinical evaluation and is not based exclusively on the presence of any ECG abnormality.  In the present study, the reported electrocardiographic abnormalities included both minor and major alterations, whereas the clinical classification recorded in the medical charts may not have considered certain isolated minor abnormalities sufficient to reclassify a patient from the indeterminate to the cardiac form.  However, considering this limitation and aiming to avoid possible misinterpretations or inconsistencies, we decided to remove this information from the manuscript, thereby making the study more coherent and robust. We thank the reviewer for the pertinent comment and the opportunity to clarify this point. |
| 4. Duplicate paragraph on the treatment follow-up protocol (Results and Discussion). Reviewer 2 explicitly flagged (lines 271–276 of the original submission) that the description of the treatment follow-up protocol was unnecessarily repeated, and the authors’ response stated that the wording had been revised to avoid repetition. In the R1 manuscript, however, the protocol, encompassing patient counseling on adverse drug reactions, weekly telephone follow-up, prompt clinical management, and scheduled laboratory assessments at 30 days and end of treatment, is described in essentially identical terms in both the Results section and the Discussion section. This constitutes redundant text and must be resolved: the procedural description belongs in the Results (or Methods), and the Discussion should refer back to it analytically rather than restating it verbatim. | We apologize. We removed the duplicated excerpt from the Discussion section. |
