# Supplementary material for: Chagas Disease in northern Minas Gerais: Clinical and epidemiological features of patients at a pioneering specialized outpatient service
Source: PLoS Negl Trop Dis. 2026 Jul 6;20(7):e0014488. doi: 10.1371/journal.pntd.0014488 (PMC13354082; doi:10.1371/journal.pntd.0014488)
Supplement: Attachment — Submitted filename: letter_reviewers.docx [file pntd.0014488.s002.docx]

Dear Editor and revierwers of the Plos Neglected Disease,

Initially, on behalf of all the authors of this manuscript, I would like to thank you for all contributions they have made for our manuscript. We do believe that the criticisms and suggestions were fundamental for improvement of our study.

We described the criticisms and suggestions reported by each reviewer followed by author´s answers and comments about them. All changes that have been made in the manuscript by authors were highlighted in the text of the manuscript in order to facilitate their identification.

**MANDATORY CHANGES FOR ACCEPTANCE:**

| **Comments** | **Author´s answers** |
| --- | --- |
| Methodology Clarification: Reviewers requested more details about data collection. Please provide additional information like a clearer presentation of treatment eligibility and adherence, and a clearer description of the type of adverse reactions observed, their frequency and severity and specify how many cases led to treatment interruption. Details about the laboratoty tests used, explicitly list which laboratory exams were requested  Statistical Analysis: There was a request for comparative analysis between: Patients who completed treatment, Patients who discontinued or were lost to follow-up, Estimating odds of treatment completion based on demographic or clinical characteristics would provide valuable insight into determinants of adherence and strengthen the manuscript’s implications  Results: You need to describe more details about patiens like median/mean age, sex distribution, proportion of indeterminate vs cardiac forms, proportion treated etiologically   We look forward to receiving the revised version of the manuscript. If you have any questions regarding the requested revisions, please do not hesitate to reach out | We appreciate the suggestions, as we believe that by incorporating them, the manuscript has become more robust and interesting. We have carefully reviewed everything and included all the suggestions. |

**JOURNAL REQUIREMENTS:**

| Comentario ingles | Resposta ingles |
| --- | --- |
| 1) Please ensure that the CRediT author contributions listed for every co-author are completed accurately and in full. At this stage, the following Authors/Authors require contributions: Luciano Freitas Fernandes, Ester Cerdeira Sabino, Sâmara Fernandes Leite, Mayra Domingues Cardoso, Ana Julia Torres, Ana Clara Jesus, Ana Beatriz Cardoso, Dardiane Santos Cruz, Ariela Mota Ferreira, and Israel Molina. Please ensure that the full contributions of each author are acknowledged in the "Add/Edit/Remove Authors" section of our submission form. The list of CRediT author contributions may be found here: https://journals.plos.org/plosntds/s/authorship#loc-author-contributions | The information has been verified. |
| 2) We do not publish any copyright or trademark symbols that usually accompany proprietary names, eg ©,  ®, or TM  (e.g. next to drug or reagent names). Therefore please remove all instances of trademark/copyright symbols throughout the text, including: - ® on page: 7. | The symbol has been removed. We appreciate the review. |
| 3) Thank you for including an Ethics Statement for your study. Please include: i) A statement that formal consent was obtained (must state whether verbal/written) OR the reason consent was not obtained (e.g. anonymity). NOTE: If child participants, the statement must declare that formal consent was obtained from the parent/guardian.]. | Since this study is based on secondary data, the requirement for ICF is waived. This information was included in the manuscript. |
| 4) Please upload a copy of Figure 3 which you refer to in your text on page 8. Or, if the figure is no longer to be included as part of the submission please remove all reference to it within the text. | Figure included. Thank you for your review. |
| 5) Tables should not be uploaded as individual files. Please remove these files and include the Tables in your manuscript file as editable, cell-based objects. For more information about how to format tables, see our guidelines: https://journals.plos.org/plosntds/s/tables | We adjusted the formatting to keep the table within the manuscript. |
| 6) Some material included in your submission may be copyrighted. According to PLOSu2019s copyright policy, authors who use figures or other material (e.g., graphics, clipart, maps) from another author or copyright holder must demonstrate or obtain permission to publish this material under the Creative Commons Attribution 4.0 International (CC BY 4.0) License used by PLOS journals. Please closely review the details of PLOSu2019s copyright requirements here: PLOS Licenses and Copyright. If you need to request permissions from a copyright holder, you may use PLOS's Copyright Content Permission form. Please respond directly to this email and provide any known details concerning your material's license terms and permissions required for reuse, even if you have not yet obtained copyright permissions or are unsure of your material's copyright compatibility. Once you have responded and addressed all other outstanding technical requirements, you may resubmit your manuscript within Editorial Manager.  Potential Copyright Issues: i) Figure 2. Please (a) provide a direct link to the base layer of the map (i.e., the country or region border shape) and ensure this is also included in the figure legend; and (b) provide a link to the terms of use / license information for the base layer image or shapefile. We cannot publish proprietary or copyrighted maps (e.g. Google Maps, Mapquest) and the terms of use for your map base layer must be compatible with our CC BY 4.0 license. Note: if you created the map in a software program like R or ArcGIS, please locate and indicate the source of the basemap shapefile onto which data has been plotted. If your map was obtained from a copyrighted source please amend the figure so that the base map used is from an openly available source. Alternatively, please provide explicit written permission from the copyright holder granting you the right to publish the material under our CC BY 4.0 license. If you are unsure whether you can use a map or not, please do reach out and we will be able to help you. The following websites are good examples of where you can source open access or public domain maps: * U.S. Geological Survey (USGS) - All maps are in the public domain. (http://www.usgs.gov) * PlaniGlobe - All maps are published under a Creative Commons license so please cite u201cPlaniGlobe, http://www.planiglobe.com, CC BY 2.0u201d in the image credit after the caption. (http://www.planiglobe.com/?lang=enl) * Natural Earth - All maps are public domain. (http://www.naturalearthdata.com/about/terms-of-use/). | We appreciate the editorial team for this observation. Figure 2 was created using QGIS software, and the basemap layer was obtained from the Brazilian Institute of Geography and Statistics (IBGE, 2023;<https://www.ibge.gov.br/geociencias/downloads-geociencias.html>), wich provides public accessible data compatible with the CC BY 4.0 license. The source and licensing information have been included in the figure legend. |
| 7) We note that you have indicated that there are restrictions to data sharing for this study. PLOS only allows data to be available upon request if there are legal or ethical restrictions on sharing data publicly. For more information on unacceptable data access restrictions, please see https://journals.plos.org/plosntds/s/data-availability#loc-unacceptable-data-access-restrictions. Before we proceed with your manuscript, please address the following prompts: a) If there are ethical or legal restrictions on sharing a de-identified data set, please explain them in detail (e.g., data contain potentially identifying or sensitive patient information, data are owned by a third-party organization, etc.) and who has imposed them (e.g., a Research Ethics Committee or Institutional Review Board, etc.). Please also provide contact information for a data access committee, ethics committee, or other institutional body to which data requests may be sent. b) If there are no restrictions, please upload the minimal anonymized data set necessary to replicate your study findings to a stable, public repository and provide us with the relevant URLs, DOIs, or accession numbers. For a list of recommended repositories, please see https://journals.plos.org/plosone/s/recommended-repositories. You also have the option of uploading the data as Supporting Information files, but we would recommend depositing data directly to a data repository if possible. We will update your Data Availability statement on your behalf to reflect the information you provide. | We apologize for this. The data will be made available upon request. The study uses secondary data from a healthcare institution. For local ethical approval, a confidentiality agreement was requested. Therefore, the database will be made available to anyone who also makes this commitment. We consulted the PLOS policy which states "This data policy does not overrule local regulations, legislation or ethical frameworks". Therefore, we request this consideration, if possible. |

**REVIEWERS' COMMENTS:**

**METHODS**

**Reviewer #1:**

| **Comments** | **Author´s answers** |
| --- | --- |
| See Summary and General Comments section | Thank you. We have verified it as requested. |

**Reviewer #2:**

| **Comments** | **Author´s answers** |
| --- | --- |
| Treatment indication and adherence analysis; Given that the central argument of the manuscript is that a specialized clinical approach may improve care and adherence, a more detailed analysis of treatment indication and completion rates is essential. | We appreciate the suggestion. Information about the clinical approach and treatment completion rates has been included. |
| The manuscript would benefit from a clearer presentation of treatment eligibility and adherence: Among all patients evaluated, how many met national guideline criteria for etiological treatment? Of those eligible, how many initiated treatment? | Information about criteria adopted by the outpatient clinic on patients' treatment eligibility was included. Therefore, all those who met the criteria started the treatment at the outpatient clinic, that is 309 patients. |
| How many interrupted treatment? | This information was included in the results for patients treated by the outpatient clinic to the results section and the Fig 4. Since the research uses secondary data from medical records, it limits the collection of information from patients treated externally. In other words, we can only obtain information related to the treatment and follow-up provided by the service. |
| How many completed at least 30 days? How many completed the full recommended regimen? | This information was included to all patients with treatment information in their medical records, even if the treatment was not performed at the outpatient clinic.  Regarding the completion of the recommended regimen, we did not have access to the prescription of all of them, as part was treated previously or in another service. Therefore, we calculate the minimum 60 days of treaty, as recommended by the PCDT. We have included this information in the manuscript  In a second phase, we analyzed the patients treated by the outpatient clinic, for whom we have all the information. We added this analysis to the manuscript. |
| Detailed description of adverse reactions Adverse reactions are a critical issue in Chagas disease management, as fear of side effects often influences both physician prescribing behavior and patient adherence.  The manuscript should: i) Provide a clearer description of the type of adverse reactions observed (e.g., dermatologic, neurological, gastrointestinal). ii) Report their frequency and severity. iii)C larify whether symptomatic treatment was required. iv) Specify how many cases led to treatment interruption. (This information is particularly important to contextualize the feasibility and safety of broader treatment strategies in chronic CD). | We appreciate the suggestion. The information about type of reaction, frequency, occurrence and interruption were included in the FIG 3 and table 2.  Regarding the information about symptomatic treatment, it was not possible to obtain due to the limitation of data collection, as we used secondary data from medical records. Thus, it was not possible to systematize and standardize the requested information. |
| Although the study is presented as descriptive, some additional analyses would substantially enhance its impact:  A more detailed characterization of patient profiles, including medical history and family history of CD.  OR  A comparative analysis between: Patients who completed treatment, Patients who discontinued or were lost to follow-up, Estimating odds of treatment completion based on demographic or clinical characteristics would provide valuable insight into determinants of adherence and strengthen the manuscript’s implications. | We appreciate the suggestions. We have included valuable information in the manuscript based on the reviewers’ suggestions.  we analyzed the patients treated by the outpatient clinic, for whom we have all the information. We added this analysis to the manuscript. |

**RESULTS**

**Reviewer #1:**

| **Comments** | **Author´s answers** |
| --- | --- |
| See Summary and General Comments section | Thank you. We have verified it as requested. |

**Reviewer #2:**

| **Comments** | **Author´s answers** |
| --- | --- |
| The tables are generally clear and well structured. However:  Figures should be fully presented in English.  Figure 1 requires clarification regarding whether the number of visits corresponds to number of patients or total consultations (as repeated visits may inflate counts). | We appreciate the comment. We edited the figures to make them correct.  Figure 1 refers to the number of patients. This information has been clarified in the figure legend. |
| Table 2 would benefit from a comparison between treated vs untreated patients. | A column with the suggested information has been added. |
| A flow chart describing patient inclusion, treatment indication, initiation, completion, and loss to follow-up would substantially improve clarity. | A flowchart with the information has been included (Figure 3). |
| In the text:  L 203 – L 212 Please double check the results as they are repeated phrases and different results of adverse drug reactions. | We apologize, the incorrect sentence has been removed from the manuscript. |

**CONCLUSIONS**

**Reviewer #1:**

| **Comments** | **Author´s answers** |
| --- | --- |
| See Summary and General Comments section | Thank you. We have verified it as requested. |

**Reviewer #2:**

| **Comments** | **Author´s answers** |
| --- | --- |
| It is interesting to understant the clincial profile of the patients but I would focus on i) why the chagas diseases clinics are innovative, please describe better the patients follow up and the patients way from the primary care until the treatment. Does all of the patients were fully assessed (EKG, Gastrintestinal Chagas diseases screening?). | We appreciate the comments. Regarding innovation, the focus is on the structured follow-up protocol for treatment, which combines patient education, weekly phone calls, immediate clinical management, and scheduled consultations. This approach has improved patient adherence. We described the patient pathway for accessing the outpatient clinic |
| ii) Please make a clear statement, does a CD specific clinic is better than usual care? Why? Do your results say that? I think patients and CD patients societies have been addressing the need for a special care for ages, and know that it is happen you could state it clearly | The standard of care would typically be provided within primary healthcare in Brazil. However, at this level of care, there is no established care pathway, protocols, or flowcharts to support healthcare professionals. There is also a noticeable sense of uncertainty and lack of knowledge among doctors, even in endemic regions (DOI: 10.34019/1809-8363.2023.v26.38290; 10.1371/journal.pntd.0008782). The research team behind this manuscript has previously attempted to provide structured training for primary healthcare professionals. However, a significant challenge remains the high turnover of staff. In this context, a specialized clinic can concentrate on monitoring disease progression and delivering treatment safely and effectively, ultimately ensuring higher-quality patient care.  . |

**SUMMARY AND GENERAL COMMENTS**

**Reviewer #1:**

| **Comments** | **Author´s answers** |
| --- | --- |
| MAJOR COMMENTS |  |
| The study aims to describe the clinical and epidemiological profile of patients; however, the abstract reports almost exclusively service utilization metrics (number of patients, municipalities, demand growth). No clinical or epidemiological characteristics (age, sex, clinical forms, comorbidities, ECG findings, treatment proportions) are presented. Include at least key clinical and epidemiological descriptors in the Results section of the abstract (e.g., median/mean age, sex distribution, proportion of indeterminate vs cardiac forms, proportion treated etiologically). | We thank the reviewer for the comment. The relevant information suggested has been incorporated into the abstract. |
| Two different and conflicting sets of numbers are presented. This is a critical issue. Clarify and correct these numbers, ensure consistency throughout text, tables, and figures, and explain denominators clearly. Lines 203–207: 302 prescriptions, 214 adverse reactions Lines 208–212: 386 prescriptions, 217 adverse reactions | We appreciate the reviewer for the careful review. This was an error and now has been duly corrected. |
| Line 218 states that laboratory monitoring was performed at 30 days and end of treatment, but no tests are specified. Explicitly list which laboratory exams were requested (e.g., complete blood count, liver enzymes, renal function, others), and justify based on national or international guidelines. | Laboratory monitoring includes clinical examinations, complete blood count, renal function tests, and assessment of liver enzymes, in accordance with the Clinical Protocol and Therapeutic Guidelines for Chagas Disease. This information has been incorporated. |
| The text states “Medication use was also assessed by therapeutic class, showing antihypertensives as the most frequently prescribed (Figure 3)” but Figure 3 is not present in the submission package. Either include Figure 3 or remove the citation and describe medication data only in text or table. | Figure included. Thank you for your review. |
| Language and formatting issues in figures. Figure 2 contains Portuguese legend and labels. Figure 1 shows months in lowercase and mixed formatting. Translate all figure elements into English and standardize month abbreviations in capital letters (e.g., OCT-22, NOV-22). | The suggestions have been incorporated. |
| The Discussion states concern about persistence of congenital or vectorial transmission based on one 4-year-old patient. Rephrase cautiously, indicating this as a hypothesis or signal rather than implying confirmed ongoing transmission, unless supporting epidemiological evidence is provided. | We appreciate your comments. The passage has been revised to retain the statement as a hypothesis, as suggested. |
| MINOR COMMENTS |  |
| Abstract and text • Italicize Trypanosoma cruzi at all occurrences. • Avoid repeating “descriptive cross-sectional” multiple times; streamline language. • Replace “Medium 58.7” in Table 2 with “Mean 58.7” (or “Median” if appropriate). | We appreciate your comment. The suggestions have been incorporated. |
| Methods • Clarify how missing data were handled (some variables do not sum to n = 931). | We thank the reviewer for the careful reading and valuable observation. Missing data were treated as losses in the analysis. As this study was based on secondary data, the occurrence of incomplete information was expected. Efforts were made to retrieve as much information as possible from the available records; however, in some cases, the missing data could not be obtained. This information has been incorporated into the manuscript. |
| • Briefly describe quality control for data extraction from paper vs electronic records. | We thank the reviewer for the observation. This information has been incorporated into the manuscript.In the outpatient clinic, a standardized template is used to collect patient information, except for medical progress notes, which facilitates standardization and, consequently, improves data quality. The researchers responsible for data collection were previously trained and calibrated in data extraction procedures. In cases where the information recorded did not follow the expected standard or raised interpretative uncertainties, the records were reviewed and discussed jointly by all authors to ensure greater consistency and reliability of the collected data. |
| • Specify whether ECG interpretation followed standardized criteria or cardiologist report. | Incluímos essa informação na seção de métodos. Foi pelo laudo disponibilizado pelo paciente durante a consulta médica |
| Results • Consider adding a short paragraph summarizing the most important clinical profile findings before moving to service organization. | We sincerely thank the reviewer for this thoughtful suggestion. However, we have chosen to maintain the current structure of the manuscript, beginning with the description of the healthcare service. We believe that this organization provides the necessary context for an appropriate interpretation of the clinical findings presented subsequently, contributing to a more logical and coherent flow for the reader.  We nonetheless value this comment and hope that our rationale will be taken into consideration |
| • Report adverse drug reaction proportion as percentage in addition to absolute numbers. | The suggestion have been incorporated. |
| Discussion • Expand comparison with other Brazilian or Latin American experiences of specialized Chagas services, if available. • Add brief discussion of sustainability and scalability challenges (human resources, funding, integration with PHC). | We thank the reviewer for the suggestion. The Discussion section has been restructured as recommended. |
| Tables • Table 2 footnote “* variation of n=931” should be rewritten as “* Denominator varies due to missing data”. • Ensure consistent decimal formatting (e.g., 64.1% vs 64,1%). | We appreciate the comments. The suggestions have been incorporated. |
| Technical / Editorial • Minor grammatical polishing throughout the manuscript is recommended. Therefore, I suggest that the text be reviewed by a native English speaker. • Ensure consistent capitalization of “Chagas disease” and “chronic Chagas cardiomyopathy”. • Confirm that SciELO Data repository link is functional and accessible. | We thank the reviewer for the suggestions, which have been fully incorporated. |

**Reviewer #2:**

| **Comments** | **Author´s answers** |
| --- | --- |
| Please double check the results as they are repeated phrases and different results of adverse drug reactions. | We thank the reviewer for the comment. The necessary revisions and corrections have been made. |
| No data about the type of adverse reactions or the symptomatic treatment proposed | Adverse drug reactions datas have been incorporated into the Results section, and a flow diagram has been constructed (Figure 3 and table 2). Regarding symptomatic treatment, information about the protocol adopted by the service for the management of adverse reactions has been included. |
| L257 Treatment prescription is based on the PCDT? Why the other patients were not treated? It will be interesting in a more extense data presentation about it | We thank the reviewer for the comment. The treatment is based on the PCDT, and this information has been incorporated into the manuscript. A more detailed presentation of these data will be considered in a subsequent manuscript currently being developed by the authors, whose primary objective will be to include comprehensive data on treatment. We believe that this topic warrants dedicated investigation, given the existing gap in the literature. |
| L 268 It is not clear to me if this number is related to the patients in the current serie or on the literature, attention adverse events = adverse reactions? | The cited number refers to the patients included in the study. The wording has been revised to improve clarity, and the terminology has been standardized. |
| L 271 – L 275 the patients follow up should be clearly described in the methods (patient’s follow up) and it is not necessary to repeat in the results L 276 – again the same information about patients follow up | We thank the reviewer for the comment. The wording has been revised to avoid repetition, in accordance with the suggestions of all reviewers. |
