# Supplementary material for: Chagas Disease in northern Minas Gerais: Clinical and epidemiological features of patients at a pioneering specialized outpatient service
Source: PLoS Negl Trop Dis. 2026 Jul 6;20(7):e0014488. doi: 10.1371/journal.pntd.0014488 (PMC13354082; doi:10.1371/journal.pntd.0014488)
Supplement: Peer review history

# Peer review: Decision Letter 0

**DOI:** 10.1371/journal.pntd.0014488.r001

**Authors:** Deborah Bittencourt Mothé Fraga, Susan Madison-Antenucci

22 Feb 2026

PNTD-D-26-00098

Chagas disease in northern Minas Gerais: clinical and epidemiological features of patients at a pioneering specialized outpatient service

PLOS Neglected Tropical Diseases

Dear Dr. Fernandes,

Thank you for submitting your manuscript to PLOS Neglected Tropical Diseases. After careful consideration, we feel that it has merit but does not fully meet PLOS Neglected Tropical Diseases's publication criteria as it currently stands. Therefore, we invite you to submit a revised version of the manuscript that addresses the points raised during the review process.

Please submit your revised manuscript within by Apr 23 2026 11:59PM. If you will need more time than this to complete your revisions, please reply to this message or contact the journal office at plosntds@plos.org. When you're ready to submit your revision, log on to https://www.editorialmanager.com/pntd/ and select the 'Submissions Needing Revision' folder to locate your manuscript file.

Please include the following items when submitting your revised manuscript:

* A letter that responds to each point raised by the editor and reviewer(s). You should upload this letter as a separate file labeled 'Response to Reviewers'. This file does not need to include responses to any formatting updates and technical items listed in the 'Journal Requirements' section below.

* A marked-up copy of your manuscript that highlights changes made to the original version. You should upload this as a separate file labeled 'Revised Manuscript with Track Changes'.

* An unmarked version of your revised paper without tracked changes. You should upload this as a separate file labeled 'Manuscript'.

If you would like to make changes to your financial disclosure, competing interests statement, or data availability statement, please make these updates within the submission form at the time of resubmission. Guidelines for resubmitting your figure files are available below the reviewer comments at the end of this letter.

We look forward to receiving your revised manuscript.

Kind regards,

Deborah Bittencourt Mothé Fraga, Ph.D.

Guest Editor

PLOS Neglected Tropical Diseases

Susan Madison-Antenucci

Section Editor

PLOS Neglected Tropical Diseases

Shaden Kamhawi

co-Editor-in-Chief

PLOS Neglected Tropical Diseases

orcid.org/0000-0003-4304-636XX

Paul Brindley

co-Editor-in-Chief

PLOS Neglected Tropical Diseases

orcid.org/0000-0003-1765-0002

**Additional Editor Comments:**

Dear Author,

Thank you for your submission to PLOS Neglected Tropical Diseases. After peer review, the editorial decision is "acceptance pending major revisions." We appreciate your study’s contribution to the field and outline the following points that need to be addressed before final acceptance.

Mandatory Changes for Acceptance:

Methodology Clarification: Reviewers requested more details about data collection. Please provide additional information like a clearer presentation of treatment eligibility and adherence, and a clearer description of the type of adverse reactions observed, their frequency and severity and specify how many cases led to treatment interruption. Details about the laboratoty tests used, explicitly list which laboratory exams were requested

Statistical Analysis: There was a request for comparative analysis between:

Patients who completed treatment, Patients who discontinued or were lost to follow-up,

Estimating odds of treatment completion based on demographic or clinical characteristics would provide valuable insight into determinants of adherence and strengthen the manuscript’s implications

Results: You need to describe more details about patiens like median/mean age, sex distribution, proportion of indeterminate vs cardiac forms, proportion treated etiologically

We look forward to receiving the revised version of the manuscript. If you have any questions regarding the requested revisions, please do not hesitate to reach out

**Journal Requirements:**

If the reviewer comments include a recommendation to cite specific previously published works, please review and evaluate these publications to determine whether they are relevant and should be cited. There is no requirement to cite these works unless the editor has indicated otherwise.

1) Please ensure that the CRediT author contributions listed for every co-author are completed accurately and in full.

At this stage, the following Authors/Authors require contributions: Luciano Freitas Fernandes, Ester Cerdeira Sabino, Sâmara Fernandes Leite, Mayra Domingues Cardoso, Ana Julia Torres, Ana Clara Jesus, Ana Beatriz Cardoso, Dardiane Santos Cruz, Ariela Mota Ferreira, and Israel Molina. Please ensure that the full contributions of each author are acknowledged in the "Add/Edit/Remove Authors" section of our submission form.

The list of CRediT author contributions may be found here: https://journals.plos.org/plosntds/s/authorship#loc-author-contributions

2) We do not publish any copyright or trademark symbols that usually accompany proprietary names, eg ©,  ®, or TM  (e.g. next to drug or reagent names). Therefore please remove all instances of trademark/copyright symbols throughout the text, including:

- ® on page: 7.

3) Thank you for including an Ethics Statement for your study. Please include:

i) A statement that formal consent was obtained (must state whether verbal/written) OR the reason consent was not obtained (e.g. anonymity). NOTE: If child participants, the statement must declare that formal consent was obtained from the parent/guardian.].

4) Please upload a copy of Figure 3 which you refer to in your text on page 8. Or, if the figure is no longer to be included as part of the submission please remove all reference to it within the text.

5) Tables should not be uploaded as individual files. Please remove these files and include the Tables in your manuscript file as editable, cell-based objects. For more information about how to format tables, see our guidelines:

https://journals.plos.org/plosntds/s/tables

6) Some material included in your submission may be copyrighted. According to PLOSu2019s copyright policy, authors who use figures or other material (e.g., graphics, clipart, maps) from another author or copyright holder must demonstrate or obtain permission to publish this material under the Creative Commons Attribution 4.0 International (CC BY 4.0) License used by PLOS journals. Please closely review the details of PLOSu2019s copyright requirements here: PLOS Licenses and Copyright. If you need to request permissions from a copyright holder, you may use PLOS's Copyright Content Permission form.

Please respond directly to this email and provide any known details concerning your material's license terms and permissions required for reuse, even if you have not yet obtained copyright permissions or are unsure of your material's copyright compatibility. Once you have responded and addressed all other outstanding technical requirements, you may resubmit your manuscript within Editorial Manager.

Potential Copyright Issues:

i) Figure 2. Please (a) provide a direct link to the base layer of the map (i.e., the country or region border shape) and ensure this is also included in the figure legend; and (b) provide a link to the terms of use / license information for the base layer image or shapefile. We cannot publish proprietary or copyrighted maps (e.g. Google Maps, Mapquest) and the terms of use for your map base layer must be compatible with our CC BY 4.0 license.

Note: if you created the map in a software program like R or ArcGIS, please locate and indicate the source of the basemap shapefile onto which data has been plotted.

If your map was obtained from a copyrighted source please amend the figure so that the base map used is from an openly available source. Alternatively, please provide explicit written permission from the copyright holder granting you the right to publish the material under our CC BY 4.0 license.

If you are unsure whether you can use a map or not, please do reach out and we will be able to help you. The following websites are good examples of where you can source open access or public domain maps:

* U.S. Geological Survey (USGS) - All maps are in the public domain. (http://www.usgs.gov)

* PlaniGlobe - All maps are published under a Creative Commons license so please cite u201cPlaniGlobe, http://www.planiglobe.com, CC BY 2.0u201d in the image credit after the caption. (http://www.planiglobe.com/?lang=enl)

* Natural Earth - All maps are public domain. (http://www.naturalearthdata.com/about/terms-of-use/).

7) We note that you have indicated that there are restrictions to data sharing for this study. PLOS only allows data to be available upon request if there are legal or ethical restrictions on sharing data publicly. For more information on unacceptable data access restrictions, please see https://journals.plos.org/plosntds/s/data-availability#loc-unacceptable-data-access-restrictions.

Before we proceed with your manuscript, please address the following prompts:

a) If there are ethical or legal restrictions on sharing a de-identified data set, please explain them in detail (e.g., data contain potentially identifying or sensitive patient information, data are owned by a third-party organization, etc.) and who has imposed them (e.g., a Research Ethics Committee or Institutional Review Board, etc.). Please also provide contact information for a data access committee, ethics committee, or other institutional body to which data requests may be sent.

b) If there are no restrictions, please upload the minimal anonymized data set necessary to replicate your study findings to a stable, public repository and provide us with the relevant URLs, DOIs, or accession numbers. For a list of recommended repositories, please see https://journals.plos.org/plosone/s/recommended-repositories. You also have the option of uploading the data as Supporting Information files, but we would recommend depositing data directly to a data repository if possible.

We will update your Data Availability statement on your behalf to reflect the information you provide.

**Reviewers' Comments:**

Reviewer's Responses to Questions

**Key Review Criteria Required for Acceptance?**

As you describe the new analyses required for acceptance, please consider the following:

**Methods**

-Are the objectives of the study clearly articulated with a clear testable hypothesis stated?

-Is the study design appropriate to address the stated objectives?

-Is the population clearly described and appropriate for the hypothesis being tested?

-Is the sample size sufficient to ensure adequate power to address the hypothesis being tested?

-Were correct statistical analysis used to support conclusions?

-Are there concerns about ethical or regulatory requirements being met?

Reviewer #1: See Summary and General Comments section

Reviewer #2: The manuscript describes the implementation of a specialized outpatient clinic for Chagas disease (CD) in a hyperendemic region and aims to characterize the clinical and epidemiological profile of patients attending this service. The initiative is highly relevant; however, several key points should be addressed to strengthen the study and enhance its scientific contribution.

1) Treatment indication and adherence analysis; Given that the central argument of the manuscript is that a specialized clinical approach may improve care and adherence, a more detailed analysis of treatment indication and completion rates is essential.

The manuscript would benefit from a clearer presentation of treatment eligibility and adherence:

Among all patients evaluated, how many met national guideline criteria for etiological treatment?

Of those eligible, how many initiated treatment? How many interrupted treatment?

How many completed at least 30 days?

How many completed the full recommended regimen?

2) Detailed description of adverse reactions

Adverse reactions are a critical issue in Chagas disease management, as fear of side effects often influences both physician prescribing behavior and patient adherence.

The manuscript should:

i) Provide a clearer description of the type of adverse reactions observed (e.g., dermatologic, neurological, gastrointestinal).

ii) Report their frequency and severity.

iii)C larify whether symptomatic treatment was required.

iv) Specify how many cases led to treatment interruption.

(This information is particularly important to contextualize the feasibility and safety of broader treatment strategies in chronic CD).

3) Although the study is presented as descriptive, some additional analyses would substantially enhance its impact:

A more detailed characterization of patient profiles, including medical history and family history of CD.

OR

A comparative analysis between:

Patients who completed treatment, Patients who discontinued or were lost to follow-up,

Estimating odds of treatment completion based on demographic or clinical characteristics would provide valuable insight into determinants of adherence and strengthen the manuscript’s implications.

**********

**Results**

-Does the analysis presented match the analysis plan?

-Are the results clearly and completely presented?

-Are the figures (Tables, Images) of sufficient quality for clarity?

Reviewer #1: See Summary and General Comments section

Reviewer #2: The tables are generally clear and well structured. However:

Figures should be fully presented in English.

Figure 1 requires clarification regarding whether the number of visits corresponds to number of patients or total consultations (as repeated visits may inflate counts).

Table 2 would benefit from a comparison between treated vs untreated patients.

A flow chart describing patient inclusion, treatment indication, initiation, completion, and loss to follow-up would substantially improve clarity.

In the text:

L 203 – L 212 Please double check the results as they are repeated phrases and different results of adverse drug reactions.

**********

**Conclusions**

-Are the conclusions supported by the data presented?

-Are the limitations of analysis clearly described?

-Do the authors discuss how these data can be helpful to advance our understanding of the topic under study?

-Is public health relevance addressed?

Reviewer #1: See Summary and General Comments section

Reviewer #2: It is interesting to understant the clincial profile of the patients but I would focus on

i) why the chagas diseases clinics are innovative, please describe better the patients follow up and the patients way from the primary care until the treatment. Does all of the patients were fully assessed (EKG, Gastrintestinal Chagas diseases screening?).

ii) Please make a clear statement, does a CD specific clinic is better than usual care? Why? Do your results say that?

I think patients and CD patients societies have been addressing the need for a special care for ages, and know that it is happen you could state it clearly

**********

**Editorial and Data Presentation Modifications?**

Use this section for editorial suggestions as well as relatively minor modifications of existing data that would enhance clarity. If the only modifications needed are minor and/or editorial, you may wish to recommend “Minor Revision” or “Accept”.

Reviewer #1: See Summary and General Comments section

Reviewer #2: (No Response)

**********

**Summary and General Comments**

Use this section to provide overall comments, discuss strengths/weaknesses of the study, novelty, significance, general execution and scholarship. You may also include additional comments for the author, including concerns about dual publication, research ethics, or publication ethics. If requesting major revision, please articulate the new experiments that are needed.

Reviewer #1: Dear Editor,

This manuscript describes the implementation and initial experience of a pioneering specialized outpatient clinic for Chagas disease in a hyperendemic region of northern Minas Gerais. The topic is highly relevant and aligns well with the scope of PLOS Neglected Tropical Diseases, as it addresses access to care, service organization, and real-world implementation challenges for Chagas disease management. The dataset is large, the regional context is well justified, and the experience reported has potential to inform policy and practice in other endemic settings. However, the manuscript requires moderate revision to strengthen coherence between objectives and results, improve clarity and consistency of reporting, address internal inconsistencies, and enhance methodological transparency.

MAJOR COMMENTS

1. The study aims to describe the clinical and epidemiological profile of patients; however, the abstract reports almost exclusively service utilization metrics (number of patients, municipalities, demand growth). No clinical or epidemiological characteristics (age, sex, clinical forms, comorbidities, ECG findings, treatment proportions) are presented. Include at least key clinical and epidemiological descriptors in the Results section of the abstract (e.g., median/mean age, sex distribution, proportion of indeterminate vs cardiac forms, proportion treated etiologically).

2. Two different and conflicting sets of numbers are presented. This is a critical issue. Clarify and correct these numbers, ensure consistency throughout text, tables, and figures, and explain denominators clearly.

Lines 203–207: 302 prescriptions, 214 adverse reactions

Lines 208–212: 386 prescriptions, 217 adverse reactions

3. Line 218 states that laboratory monitoring was performed at 30 days and end of treatment, but no tests are specified. Explicitly list which laboratory exams were requested (e.g., complete blood count, liver enzymes, renal function, others), and justify based on national or international guidelines.

4. The text states “Medication use was also assessed by therapeutic class, showing antihypertensives as the most frequently prescribed (Figure 3)” but Figure 3 is not present in the submission package. Either include Figure 3 or remove the citation and describe medication data only in text or table.

5. Language and formatting issues in figures. Figure 2 contains Portuguese legend and labels. Figure 1 shows months in lowercase and mixed formatting. Translate all figure elements into English and standardize month abbreviations in capital letters (e.g., OCT-22, NOV-22).

6. The Discussion states concern about persistence of congenital or vectorial transmission based on one 4-year-old patient. Rephrase cautiously, indicating this as a hypothesis or signal rather than implying confirmed ongoing transmission, unless supporting epidemiological evidence is provided.

MINOR COMMENTS

Abstract and text

• Italicize Trypanosoma cruzi at all occurrences.

• Avoid repeating “descriptive cross-sectional” multiple times; streamline language.

• Replace “Medium 58.7” in Table 2 with “Mean 58.7” (or “Median” if appropriate).

Methods

• Clarify how missing data were handled (some variables do not sum to n = 931).

• Briefly describe quality control for data extraction from paper vs electronic records.

• Specify whether ECG interpretation followed standardized criteria or cardiologist report.

Results

• Consider adding a short paragraph summarizing the most important clinical profile findings before moving to service organization.

• Report adverse drug reaction proportion as percentage in addition to absolute numbers.

Discussion

• Expand comparison with other Brazilian or Latin American experiences of specialized Chagas services, if available.

• Add brief discussion of sustainability and scalability challenges (human resources, funding, integration with PHC).

Tables

• Table 2 footnote “* variation of n=931” should be rewritten as “* Denominator varies due to missing data”.

• Ensure consistent decimal formatting (e.g., 64.1% vs 64,1%).

Technical / Editorial

• Minor grammatical polishing throughout the manuscript is recommended. Therefore, I suggest that the text be reviewed by a native English speaker.

• Ensure consistent capitalization of “Chagas disease” and “chronic Chagas cardiomyopathy”.

• Confirm that SciELO Data repository link is functional and accessible.

Reviewer #2: L 203 – L 212 Please double check the results as they are repeated phrases and different results of adverse drug reactions.

No data about the type of adverse reactions or the symptomatic treatment proposed

L257 Treatment prescription is based on the PCDT? Why the other patients were not treated? It will be interesting in a more extense data presentation about it

L 268 It is not clear to me if this number is related to the patients in the current serie or on the literature, attention adverse events = adverse reactions?

L 271 – L 275 the patients follow up should be clearly described in the methods (patient’s follow up) and it is not necessary to repeat in the results

L 276 – again the same information about patients follow up

**********

PLOS authors have the option to publish the peer review history of their article (what does this mean?). If published, this will include your full peer review and any attached files.

If you choose “no”, your identity will remain anonymous but your review may still be made public.

**Do you want your identity to be public for this peer review?**  For information about this choice, including consent withdrawal, please see our Privacy Policy.

Reviewer #1: **Yes:** FRED LUCIANO NEVES SANTOS

Reviewer #2: No

**Figure resubmission:**

While revising your submission, we strongly recommend that you use PLOS’s NAAS tool (https://ngplosjournals.pagemajik.ai/artanalysis) to test your figure files. NAAS can convert your figure files to the TIFF file type and meet basic requirements (such as print size, resolution), or provide you with a report on issues that do not meet our requirements and that NAAS cannot fix.

After uploading your figures to PLOS’s NAAS tool - https://ngplosjournals.pagemajik.ai/artanalysis, NAAS will process the files provided and display the results in the "Uploaded Files" section of the page as the processing is complete. If the uploaded figures meet our requirements (or NAAS is able to fix the files to meet our requirements), the figure will be marked as "fixed" above. If NAAS is unable to fix the files, a red "failed" label will appear above. When NAAS has confirmed that the figure files meet our requirements, please download the file via the download option, and include these NAAS processed figure files when submitting your revised manuscript.

**Reproducibility:**

To enhance the reproducibility of your results, we recommend that authors of applicable studies deposit laboratory protocols in protocols.io, where a protocol can be assigned its own identifier (DOI) such that it can be cited independently in the future. Additionally, PLOS ONE offers an option to publish peer-reviewed clinical study protocols. Read more information on sharing protocols at https://plos.org/protocols?utm_medium=editorial-email&utm_source=authorletters&utm_campaign=protocols

# Author comment: Author response to Decision Letter 1

**DOI:** 10.1371/journal.pntd.0014488.r002

23 Apr 2026

Attachment Submitted filename: letter_reviewers.docx

# Peer review: Decision Letter 1

**DOI:** 10.1371/journal.pntd.0014488.r003

**Authors:** Deborah Bittencourt Mothé Fraga, Susan Madison-Antenucci

31 May 2026

PNTD-D-26-00098R1Doença de Chagas no norte de Minas Gerais: características clínicas e epidemiológicas de pacientes em um serviço ambulatorial especializado pioneiro. PLOS Neglected Tropical Diseases

Dear Dr. Fernandes,

Thank you for submitting your manuscript to PLOS Neglected Tropical Diseases. After careful consideration, we feel that it has merit but does not fully meet PLOS Neglected Tropical Diseases's publication criteria as it currently stands. Therefore, we invite you to submit a revised version of the manuscript that addresses the points raised during the review process.

Please submit your revised manuscript by Jun 30 2026 11:59PM. If you will need more time than this to complete your revisions, please reply to this message or contact the journal office at plosntds@plos.org. When you're ready to submit your revision, log on to https://www.editorialmanager.com/pntd/ and select the 'Submissions Needing Revision' folder to locate your manuscript file.

Please include the following items when submitting your revised manuscript:

* A letter that responds to each point raised by the editor and reviewer(s). You should upload this letter as a separate file labeled 'Response to Reviewers'. This file does not need to include responses to any formatting updates and technical items listed in the 'Journal Requirements' section below.

* A marked-up copy of your manuscript that highlights changes made to the original version. You should upload this as a separate file labeled 'Revised Manuscript with Track Changes'.

* An unmarked version of your revised paper without tracked changes. You should upload this as a separate file labeled 'Manuscript'.

If you would like to make changes to your financial disclosure, competing interests statement, or data availability statement, please make these updates within the submission form at the time of resubmission. Guidelines for resubmitting your figure files are available below the reviewer comments at the end of this letter.

As the corresponding author, your ORCID iD is verified in the submission system and will appear in the published article. PLOS supports the use of ORCID, and we encourage all coauthors to register for an ORCID iD and use it as well. Please encourage your coauthors to verify their ORCID iD within the submission system before final acceptance, as unverified ORCID iDs will not appear in the published article. Only the individual author can complete the verification step; PLOS staff cannot verify ORCID iDs on behalf of authors. We look forward to receiving your revised manuscript. Kind regards, Deborah Bittencourt Mothé Fraga, Ph.D.

Guest Editor

PLOS Neglected Tropical Diseases Susan Madison-AntenucciSection EditorPLOS Neglected Tropical Diseases

Shaden Kamhawi

co-Editor-in-Chief

PLOS Neglected Tropical Diseases

orcid.org/0000-0003-4304-636XX

Paul Brindley

co-Editor-in-Chief

PLOS Neglected Tropical Diseases

orcid.org/0000-0003-1765-0002

**Additional Editor Comments :**  Dear Authors,

Thank you for submitting the revised version and for the dedication you have shown throughout this process.

We have good news: the reviewers acknowledged that the revision was careful and thorough. The following improvements were highlighted as satisfactory: addition of clinical and epidemiological descriptors to the abstract; correction of the numerical inconsistency in the treatment data; inclusion of the missing flowchart (Figure 3); specification of laboratory monitoring tests; correction of language and formatting in the figures; and expansion of the Discussion to address sustainability aspects and comparative experiences.

There are, however, five points raised by the reviewers that still require attention before final acceptance: two are carry-over issues from the previous round that remain unresolved, and three are new observations identified in the current version.

The editorial decision is **minor revision**. We are confident that the necessary adjustments are minor and that the manuscript is close to acceptance. Please consult the reviewers' reports attached and submit the corrected version along with a response letter. **Journal Requirements:**

1) Please ensure that the Title in your manuscript file and the Title provided in your online submission form are the same.

Note: If the reviewer comments include a recommendation to cite specific previously published works, please review and evaluate these publications to determine whether they are relevant and should be cited. There is no requirement to cite these works unless the editor has indicated otherwise.

**Reviewers' comments:**

Reviewer's Responses to Questions

**Key Review Criteria Required for Acceptance?**

As you describe the new analyses required for acceptance, please consider the following:

**Methods**

-Are the objectives of the study clearly articulated with a clear testable hypothesis stated?

-Is the study design appropriate to address the stated objectives?

-Is the population clearly described and appropriate for the hypothesis being tested?

-Is the sample size sufficient to ensure adequate power to address the hypothesis being tested?

-Were correct statistical analysis used to support conclusions?

-Are there concerns about ethical or regulatory requirements being met?

Reviewer #1: See Summary and General Comments

Reviewer #2: The study objectives are clearly stated and are appropriate for a descriptive epidemiological study. The primary aim was to characterize the clinical and epidemiological profile of patients attending a newly implemented referral center for Chagas disease in a hyperendemic region of Brazil. As a descriptive study, no formal hypothesis testing was planned, and therefore no specific null hypothesis was defined.

The study design is appropriate to address the stated objectives. The target population is clearly described, including all patients evaluated at the referral center during the study period, and is representative of the population of interest.

Because the study is descriptive and does not seek to test a predefined hypothesis or estimate causal effects, a formal sample size calculation was not required. The analysis included all eligible patients attending the service during the study period.

Statistical analyses were limited to descriptive statistics and exploratory analyses intended to summarize patient characteristics, service utilization, and treatment outcomes. These methods were appropriate for the study objectives and support the conclusions presented.

No concerns were identified regarding ethical or regulatory requirements. The study was approved by the appropriate Research Ethics Committee and conducted in accordance with applicable ethical and regulatory standards.

**********

**Results**

-Does the analysis presented match the analysis plan?

-Are the results clearly and completely presented?

-Are the figures (Tables, Images) of sufficient quality for clarity?

Reviewer #1: See Summary and General Comments

Reviewer #2: The direction of the odds ratios presented in Table 2 is unclear. For some variables the reported ORs appear to model treatment completion, whereas for adverse drug reactions the ORs appear to model treatment discontinuation. The authors should clarify the outcome definition used in the logistic regression analyses and verify the consistency of the reported estimates."

**********

**Conclusions**

-Are the conclusions supported by the data presented?

-Are the limitations of analysis clearly described?

-Do the authors discuss how these data can be helpful to advance our understanding of the topic under study?

-Is public health relevance addressed?

Reviewer #1: See Summary and General Comments

Reviewer #2: It is clear, the message and the discussion stated the need for specialized care for CD patients despite being responsability only of the primary care

**********

**Editorial and Data Presentation Modifications?**

Use this section for editorial suggestions as well as relatively minor modifications of existing data that would enhance clarity. If the only modifications needed are minor and/or editorial, you may wish to recommend “Minor Revision” or “Accept”.

Reviewer #1: See Summary and General Comments

Reviewer #2: (No Response)

**********

**Summary and General Comments**

Use this section to provide overall comments, discuss strengths/weaknesses of the study, novelty, significance, general execution and scholarship. You may also include additional comments for the author, including concerns about dual publication, research ethics, or publication ethics. If requesting major revision, please articulate the new experiments that are needed.

Reviewer #1: Dear Editor,

I have reviewed the revised version of the manuscript (R1) and compared it against the comments I submitted in the previous round. I commend the authors for the thoroughness of their revision: the majority of the concerns raised have been adequately addressed, and the manuscript is substantially improved relative to the original submission. The addition of clinical and epidemiological descriptors to the abstract, the resolution of the critical numerical inconsistency in the treatment data, the inclusion of the missing flowchart (Figure 3), the specification of laboratory monitoring tests, the correction of figure language and formatting, and the expansion of the Discussion to address sustainability and comparative experiences are all appropriate and satisfactory responses. Notwithstanding, I identify four remaining issues that must be corrected before acceptance. Two are carry-overs from my original review that were not resolved; two are new observations arising from the revised text. I recommend minor revision with the expectation that the four issues described above are corrected prior to acceptance.

1. “Medium” vs. “Mean” in table headers (Tables 1 and 2). In my original review, I explicitly requested that “Medium 58.7” be replaced by “Mean 58.7” (or “Median” if applicable) throughout the tables. The author response letter acknowledges this request. However, the column header in both Table 1 (“Mean Descriptive” column reads “Medium”) and Table 2 (“Age Medium 56.8 ±10.1”) still uses the incorrect term. “Medium” is not a recognized statistical descriptor; “Mean” is required. This is a straightforward typographical correction that must be made.

2. Decimal formatting inconsistency in Table 2. The request to ensure consistent decimal formatting across the manuscript (period separator, not comma) was acknowledged but not fully implemented. Table 2 of the original submission, now integrated into the main manuscript, continues to use comma notation in percentage values (e.g., 64,1%; 65,2%; 16,8%), whereas the revised text and Table 1 use period notation. All numerical values must use a uniform period decimal separator throughout.

3. Logical inconsistency between ECG findings and classification of the indeterminate form. The revised manuscript states that 69% of patients presented ECG abnormalities, with the parenthetical clarification that “any minor or major change is considered to be altered” (Discussion, R1). The manuscript simultaneously reports that the indeterminate form was the most prevalent clinical classification (47.2%). This juxtaposition creates a direct logical contradiction with the diagnostic criteria the authors themselves cite: Reference 17 (2nd Brazilian Consensus on Chagas Disease, 2015) defines the indeterminate form as requiring a normal ECG, normal chest X-ray, and normal esophageal and colonic studies. If the ECG criterion used in this study departs from the consensus, for instance, by considering only major changes as defining cardiac involvement, this must be explicitly stated in the Methods. As currently written, readers are left with an unexplained inconsistency: nearly half the cohort is classified as indeterminate, yet most of the same cohort has an abnormal ECG by the study’s own definition. The authors must add a clarifying sentence in the Methods section specifying which ECG alterations were used to reclassify a patient from indeterminate to cardiac form, with reference to the applicable guideline.

4. Duplicate paragraph on the treatment follow-up protocol (Results and Discussion). Reviewer 2 explicitly flagged (lines 271–276 of the original submission) that the description of the treatment follow-up protocol was unnecessarily repeated, and the authors’ response stated that the wording had been revised to avoid repetition. In the R1 manuscript, however, the protocol, encompassing patient counseling on adverse drug reactions, weekly telephone follow-up, prompt clinical management, and scheduled laboratory assessments at 30 days and end of treatment, is described in essentially identical terms in both the Results section and the Discussion section. This constitutes redundant text and must be resolved: the procedural description belongs in the Results (or Methods), and the Discussion should refer back to it analytically rather than restating it verbatim.

Reviewer #2: (No Response)

**********

PLOS authors have the option to publish the peer review history of their article (what does this mean?). If published, this will include your full peer review and any attached files.

If you choose “no”, your identity will remain anonymous but your review may still be made public.

**Do you want your identity to be public for this peer review?**  For information about this choice, including consent withdrawal, please see our Privacy Policy.

Reviewer #1: **Yes:** Fred Luciano Neves Santos

Reviewer #2: No

[NOTE: If reviewer comments were submitted as an attachment file, they will be attached to this email and accessible via the submission site. Please log into your account, locate the manuscript record, and check for the action link "View Attachments". If this link does not appear, there are no attachment files.]

**Figure resubmission:**

While revising your submission, we strongly recommend that you use PLOS’s NAAS tool (https://ngplosjournals.pagemajik.ai/artanalysis) to test your figure files. NAAS can convert your figure files to the TIFF file type and meet basic requirements (such as print size, resolution), or provide you with a report on issues that do not meet our requirements and that NAAS cannot fix.  After uploading your figures to PLOS’s NAAS tool - https://ngplosjournals.pagemajik.ai/artanalysis, NAAS will process the files provided and display the results in the "Uploaded Files" section of the page as the processing is complete. If the uploaded figures meet our requirements (or NAAS is able to fix the files to meet our requirements), the figure will be marked as "fixed" above. If NAAS is unable to fix the files, a red "failed" label will appear above. When NAAS has confirmed that the figure files meet our requirements, please download the file via the download option, and include these NAAS processed figure files when submitting your revised manuscript.**Reproducibility:**  To enhance the reproducibility of your results, we recommend that authors of applicable studies deposit laboratory protocols in protocols.io, where a protocol can be assigned its own identifier (DOI) such that it can be cited independently in the future. Additionally, PLOS ONE offers an option to publish peer-reviewed clinical study protocols. Read more information on sharing protocols at https://plos.org/protocols?utm_medium=editorial-email&utm_source=authorletters&utm_campaign=protocols

# Author comment: Author response to Decision Letter 2

**DOI:** 10.1371/journal.pntd.0014488.r004

3 Jun 2026

Attachment Submitted filename: Response to Reviewers.docx

# Editor report: Decision Letter 2

**DOI:** 10.1371/journal.pntd.0014488.r005

**Authors:** Deborah Bittencourt Mothé Fraga, Susan Madison-Antenucci

22 Jun 2026

Dear Dr Fernandes,

We are pleased to inform you that your manuscript 'Chagas Disease in northern Minas Gerais: clinical and epidemiological features of patients at a pioneering specialized outpatient service' has been provisionally accepted for publication in PLOS Neglected Tropical Diseases.

Before your manuscript can be formally accepted you will need to complete some formatting changes, which you will receive in a follow up email. A member of our team will be in touch with a set of requests.

Please note that your manuscript will not be scheduled for publication until you have made the required changes, so a swift response is appreciated.

IMPORTANT: The editorial review process is now complete. PLOS will only permit corrections to spelling, formatting or significant scientific errors from this point onwards. Requests for major changes, or any which affect the scientific understanding of your work, will cause delays to the publication date of your manuscript.

Should you, your institution's press office or the journal office choose to press release your paper, you will automatically be opted out of early publication. We ask that you notify us now if you or your institution is planning to press release the article. All press must be co-ordinated with PLOS.

Thank you again for supporting Open Access publishing; we are looking forward to publishing your work in PLOS Neglected Tropical Diseases.

Best regards,

Deborah Bittencourt Mothé Fraga, Ph.D.

Guest Editor

PLOS Neglected Tropical Diseases

Susan Madison-Antenucci

Section Editor

PLOS Neglected Tropical Diseases

Shaden Kamhawi

co-Editor-in-Chief

PLOS Neglected Tropical Diseases

orcid.org/0000-0003-4304-636XX

Paul Brindley

co-Editor-in-Chief

PLOS Neglected Tropical Diseases

orcid.org/0000-0003-1765-0002

***********************************************************

# Editor report: Acceptance letter

**DOI:** 10.1371/journal.pntd.0014488.r006

**Authors:** Deborah Bittencourt Mothé Fraga, Susan Madison-Antenucci

Dear Dr Fernandes,

We are delighted to inform you that your manuscript, "Chagas Disease in northern Minas Gerais: clinical and epidemiological features of patients at a pioneering specialized outpatient service," has been formally accepted for publication in PLOS Neglected Tropical Diseases.

We have now passed your article onto the PLOS Production Department who will complete the rest of the publication process. All authors will receive a confirmation email upon publication.

The corresponding author will soon be receiving a typeset proof for review, to ensure errors have not been introduced during production. Please review the PDF proof of your manuscript carefully, as this is the last chance to correct any scientific or type-setting errors. Please note that major changes, or those which affect the scientific understanding of the work, will likely cause delays to the publication date of your manuscript. Note: Proofs for Front Matter articles (Editorial, Viewpoint, Symposium, Review, etc...) are generated on a different schedule and may not be made available as quickly.

Soon after your final files are uploaded, the early version of your manuscript will be published online unless you opted out of this process. The date of the early version will be your article's publication date. The final article will be published to the same URL, and all versions of the paper will be accessible to readers.

For Research Articles, you will receive an invoice from PLOS for your publication fee after your manuscript has reached the completed accept phase. If you receive an email requesting payment before acceptance or for any other service, this may be a phishing scheme. Learn how to identify phishing emails and protect your accounts at https://explore.plos.org/phishing.

Thank you again for supporting open-access publishing; we are looking forward to publishing your work in PLOS Neglected Tropical Diseases.

Best regards,

Shaden Kamhawi

co-Editor-in-Chief

PLOS Neglected Tropical Diseases

Paul Brindley

co-Editor-in-Chief

PLOS Neglected Tropical Diseases
